# Effects of dietary organic acids and nature identical compounds on growth, immune parameters and gut microbiota of European sea bass

**DOI:** 10.1038/s41598-020-78441-9

**Published:** 2020-12-07

**Authors:** Serena Busti, Barbara Rossi, Enrico Volpe, Sara Ciulli, Andrea Piva, Federica D’Amico, Matteo Soverini, Marco Candela, Pier Paolo Gatta, Alessio Bonaldo, Ester Grilli, Luca Parma

**Affiliations:** 1 Department of Veterinary Medical Sciences, University of Bologna, Via Tolara di Sopra 50, Ozzano Emilia, 40064 Bologna, Italy; 2 Vetagro S.P.A., Via Porro 2, 42124 Reggio Emilia, Italy; 3 Unit of Microbial Ecology of Health, Department of Pharmacy and Biotechnology, University of Bologna, Via Belmeloro 6, 40126 Bologna, Italy

**Keywords:** Molecular biology, Zoology, Animal physiology, Microbiology, Microbial communities, Immunology, Cytokines, Mucosal immunology

## Abstract

A 71-day study was conducted to explore the effect of increasing dietary levels (0, 250, 500, 1000 mg kg feed^−1^; D0, D250, D500 and D1000, respectively) of a blend of microencapsulated organic acids (OA, specifically citric and sorbic acid) and nature identical compounds (NIC, specifically thymol and vanillin), on growth, intestinal immune parameters and gut microbiota (GM) of European sea bass juveniles reared under normal and subsequently suboptimal environmental conditions (high temperature, 30.0 ± 0.4 °C and low oxygen, 4.6 ± 0.6 mg L^−1^). OA and NIC did not promote growth, feed utilisation and feed intake at the inclusion tested but induced a significantly upregulation of IL-8, IL-10 and TGFβ. GM analyzed by next-generation sequencing showed that OA and NIC were able to exert prebiotic properties stimulating the development of beneficial bacteria taxa such as *Lactobacillus*,* Leuconostoc*, and *Bacillus* sp. Picrust analyses displayed a significant potential functional reconfiguration of GM promoting a decrease in inflammation-promoting and homeostatic functions at increasing OA and NIC administration. For the first time on this species the exposure to suboptimal rearing conditions was able to modify GM structure reducing LAB and increasing Proteobacteria, findings which were consistent with the inflammatory process observed at mRNA level.

## Introduction

Organic acids (OA) are organic compounds with one or more carboxyl groups, produced through the microbial fermentation of carbohydrates from various bacterial species in different metabolic pathways and conditions^1^. OA used as feed additives have a long history in improving performance and health in terrestrial livestock, especially on pigs and poultry^2–7^ while the use of these substances as growth promoters in aquafeed is recent and moderate due to limited research, but it is expected to significantly increase in the coming years^1,8,9^. As functional additives in aquafeed, OA have been reported to: (i) reduce feed pH thus increasing feed hygiene^10^, (ii) modulate gut intestinal pH and enzyme activity favouring nutrient digestibility and feed utilisation^1,11^, (iii) modulate beneficial gut microbiota by controlling enteric colonisation of opportunistic bacteria^12^, (iv) reinforce animal health^13,14^. More specifically, several studies investigated citric acid effects on various aquaculture species, such as red drum (*Sciaenops cellatus*), rainbow trout (*Oncohrynchus mykiss*), beluga sturgeon (*Huso huso*), yellowtail (*Seriola quinqueradiata*), tilapia (*Oreochromis niloticus*) and red sea bream (*Pagrus major*), reporting improvement in growth, feed intake, specific growth rate (SGR), and feed conversion ratio (FCR)^11,15–25^. However, to the best of our knowledge no studies reporting the use of citric acid as a feed additive in European sea bass have been tested.

Sorbic acid is an OA with a strong antimicrobial activity displayed at neutral pH through the inhibition of the bacterial enzymatic activity and nutrient transport system^26^.

In order to investigate its growth-promoting effect, sorbic acid has been also included in dietary blends with formic and benzoic acids and their corresponding salts, showing a relevant weight gain increase in rainbow trout^27^.

Recent studies on several fish species i.e. red hybrid tilapia (*Oreochromis niloticus* ♀ × *Oreochromis aureus* ♂)^28^, rainbow trout^12,29,30^, tilapia^31^ and zebrafish (*Danio rerio*)^28^, have also highlighted the utilization of botanicals and nature identical compounds (NIC) as aquafeed additives for their beneficial role on feed palatability, immune system and to reduce gut bacterial pathogens.

Among the most promising NIC, thymol is a monoterpene which showed positive growth promoting effects, antimicrobial activity and anti-inflammatory properties in mammals^32–34^. Similarly vanillin, beside its food flavouring functions, can exert an antimicrobial activity altering the bacterial membrane function and respiration^35–37^. To date, no studies have been conducted to test their potential effects in marine fish.

The application of OA or NIC blends in fish feed has been proposed as an optimal strategy to test their potential synergistic effects on growth performance and nutrient utilisation^1^. Infacts, the efficacy of blends could be considered higher than a single isolated molecule for the potential increased range of action sites^38^. The combination of microencapsulated citric acid, sorbic acid, thymol and vanillin, has shown positive effects in terms of performance, microbiome modulation and general health status in terrestrial livestocks^39^and more recently in the Pacific white shrimp, (*Litopenaeus vannamei*)^40^ and rainbow trout^41^. Moreover, to the best of our knowledge, a combination of OA and NIC has never been tested on marine fish species. Finally, farmed fish are subject to exposure to a large number of stressors and suboptimal conditions, some of the most common being fluctuating levels of dissolved oxygen, handling and high temperatures. For these reasons, the aims of this study were: (1) to evaluate the effects of increasing dietary level of a microencapsulated blend of citric acid, sorbic acid, thymol and vanillin on growth, feed utilisation, intestinal cytokines gene expression and gut bacterial community of sea bass; (2) to explore the effects of the aforementioned blend on intestinal cytokines gene expression and gut bacterial community of sea bass under suboptimal rearing conditions.

## Results

### Growth

Data on growth performances (final body weight and SGR), FI, FCR and survival at the end of the trial and the intermediate period, are shown in Table 1. No significant dose effect was detected in the overall and intermediate periods (Table 1). Data on biometric indices, body composition and nutritional indices are shown in Table 2. No significant dose effect in viscerosomatic index (VSI), hepatosomatic index (HSI), and condition factor (CF) was observed. Also, no significant dose effect among treatments was found in the whole body composition, protein efficiency ratio (PER), gross protein efficiency (GPE), lipid efficiency ratio (LER), and gross lipid efficiency (GLE). No mortality was observed at the end of the feeding trial and at the end of the suboptimal rearing conditions.

**Table 1:** Growth performance of European sea bass juveniles fed the experimental diets D0, D250, D500, D1000 calculated over 71 days (time range day 0–71) and at intermediate periods (time range day 0–37 and 37–71).

	Experimental diets			
	D0	D250	D500	D1000
**Time range day 0–71**				
Initial BW (g)	13.32 ± 0.2	13.13 ± 0.0	13.23 ± 0.3	13.24 ± 0.1
FBW (g)	56.74 ± 2.24	56.80 ± 2.83	55.93 ± 3.21	56.18 ± 3.91
SGR (% day^−1^)	2.04 ± 0.06	2.06 ± 0.07	2.02 ± 0.05	2.03 ± 0.11
FI (g kg ABW^−1^ day^−1^)	18.52 ± 0.30	18.10 ± 0.45	18.17 ± 0.47	18.30 ± 0.21
FCR	1.05 ± 0.00	1.02 ± 0.00	1.03 ± 0.00	1.03 ± 0.02
Survival%	100 ± 0.00	100 ± 0.00	100 ± 0.00	100 ± 0.00
**Time range day 0–37**				
Initial BW (g)	13.30 ± 0.2	13.13 ± 0.0	13.23 ± 0.3	13.24 ± 0.1
Intermediate BW (g)	35.7 ± 1.39	34.80 ± 1.61	35.56 ± 2.91	35.17 ± 0.80
SGR (% day^−1^)	2.66 ± 0.08	2.63 ± 0.12	2.67 ± 0.16	2.64 ± 0.06
FI (g kg ABW^−1^ day^−1^)	24.79 ± 0.37	23.69 ± 0.50	23.84 ± 0.80	24.07 ± 0.98
FCR	1.01 ± 0.01	0.97 ± 0.03	0.97 ± 0.02	0.98 ± 0.02
Survival%	100 ± 0.00	100 ± 0.00	100 ± 0.00	100 ± 0.00
**Time range day 37–71**				
Intermediate BW (g)	35.7 ± 1.39	34.80 ± 1.61	35.56 ± 2.91	35.17 ± 0.80
FBW (g)	56.7 ± 2.24	56.8 ± 2.83	55.938 ± 3.21	56.2 ± 3.91
SGR (%day^−1^)	1.38 ± 0.12	1.45 ± 0.13	1.36 ± 0.07	1.41 ± 0.20
FI (g kg ABW^−1^ day^−1^)	15.06 ± 1.12	15.40 ± 0.88	14.91 ± 0.14	15.23 ± 0.95
FCR	1.11 ± 0.01	1.08 ± 0.04	1.12 ± 0.04	1.11 ± 0.09
Survival%	100 ± 0.00	100 ± 0.00	100 ± 0.00	100 ± 0.00

Data are given as the mean (n = 3) ± SD. No significant differences were detected (*P* > 0.05).

*D *dose blend (citric acid 25%, sorbic acid 16.7%, thymol 1.7%, vanillin 1%, matrix of hydrogenated fats) inclusion in diet, *D0* 0 ppm, *D250* 250 ppm, *D500* 500 ppm, *D1000* 1000 ppm, *Initial BW* initial body weight, *Intermediate BW* intermediate body weight, *FBW* final body weight, *SGR* specific growth rate (% day^−1^) = 100 × (ln FBW − ln IBW)/days, *ABW* average body weight = (IBW + FBW)/2, *FI* feed intake (g kg ABW^−1^ day^−1^) = ((1000 × total ingestion)/(ABW)/(days)), *FCR* feed conversion rate = feed intake/weight gain.

**Table 2:** Biometric indices, whole body composition and nutritional indices of European sea bass fed the experimental diets D0, D250, D500, D1000 over 71 days.

	Experimental diets			
	D0	D250	D500	D1000
**Biometric indices**				
VSI	12.2 ± 1.26	12.7 ± 1.99	12.4 ± 1.50	12.3 ± 1.50
HSI	2.84 ± 0.42	2.83 ± 0.41	2.75 ± 0.47	2.81 ± 0.43
CF	1.26 ± 0.08	1.22 ± 0.06	1.25 ± 0.10	1.27 ± 0.12
**Whole body composition, %**				
Protein	16.9 ± 0.6	16.3 ± 0.3	16 ± 0.3	16.5 ± 0.6
Lipid	17.6 ± 0.7	18.9 ± 0.3	18 ± 1.6	19.1 ± 0.7
Ash	3.68 ± 0.1	3.57 ± 0.5	3.51 ± 0.3	3.51 ± 0.1
Moisture	61.7 ± 0.3	60.4 ± 0.7	60.8 ± 1.6	60.6 ± 0.3
**Nutritional indices**				
PER	1.89 ± 0.01	1.95 ± 0.01	1.92 ± 0.02	1.91 ± 0.05
GPE	31.9 ± 2.11	31.4 ± 0.87	30.1 ± 0.65	31.1 ± 0.77
LER	4.57 ± 0.02	4.71 ± 0.02	4.64 ± 0.04	4.61 ± 0.12
GLE	92.2 ± 4.04	102.3 ± 2.75	95.7 ± 8.31	101.7 ± 2.84

Biometric indices are given as the mean (n = 15) ± SD. No significant differences were detected (*P* > 0.05).

Whole body composition % are given as the mean (n = 3) ± SD.

Nutritional indices are given as the mean (n = 3) ± SD.

*VSI* Viscerosomatic index (%) = 100 × (viscera weight/FBW), *HSI* Hepatosomatic index (%) = 100 × (liver weight/FBW), *CF* Condition factor = 100 × (FBW/length^3^), *PER* Protein efficiency ratio = (FBW–IBW)/protein intake, *GPE (%)* Gross protein efficiency = 100 × [(% final body protein × FBW) − (% initial body protein × IBW)]/total protein intake fish, *LER* Lipid efficiency rate = (FBW − IBW)/lipid intake, *GLE (%)* Gross lipid efficiency = 100 × [(% final body lipid × FBW) − (% initial body lipid × IBW)]/total lipid intake fish.

### Immune and inflammatory gene expression analyses

**Figure 1:**
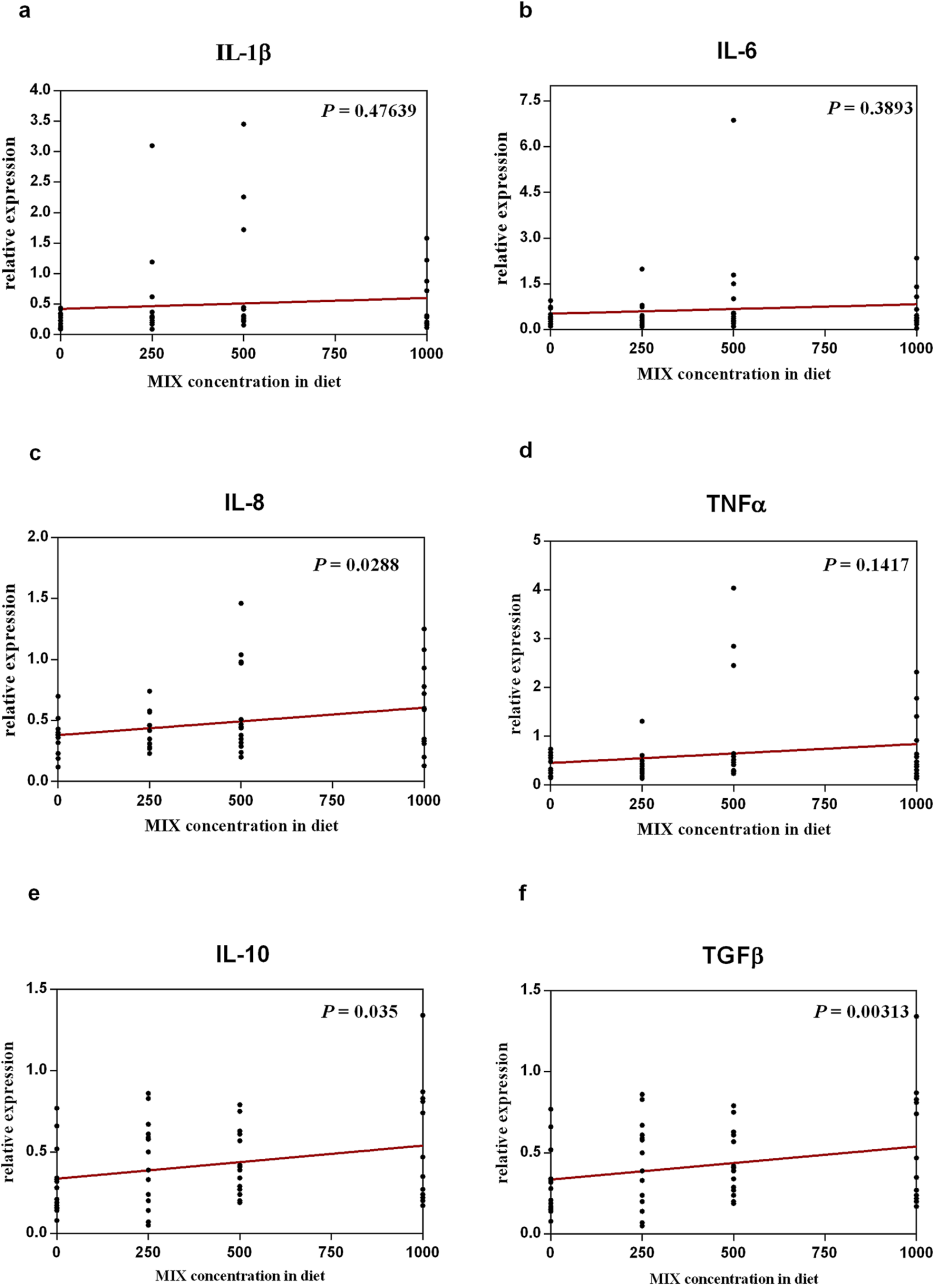
Linear regression analyses showing the effect of the dietary increasing level of OA and NIC on the relative expression of six cytokines genes (**a**–**f**) involved in the immune and inflammatory response at intestinal level at T1 (day 71).

**Figure 2:**
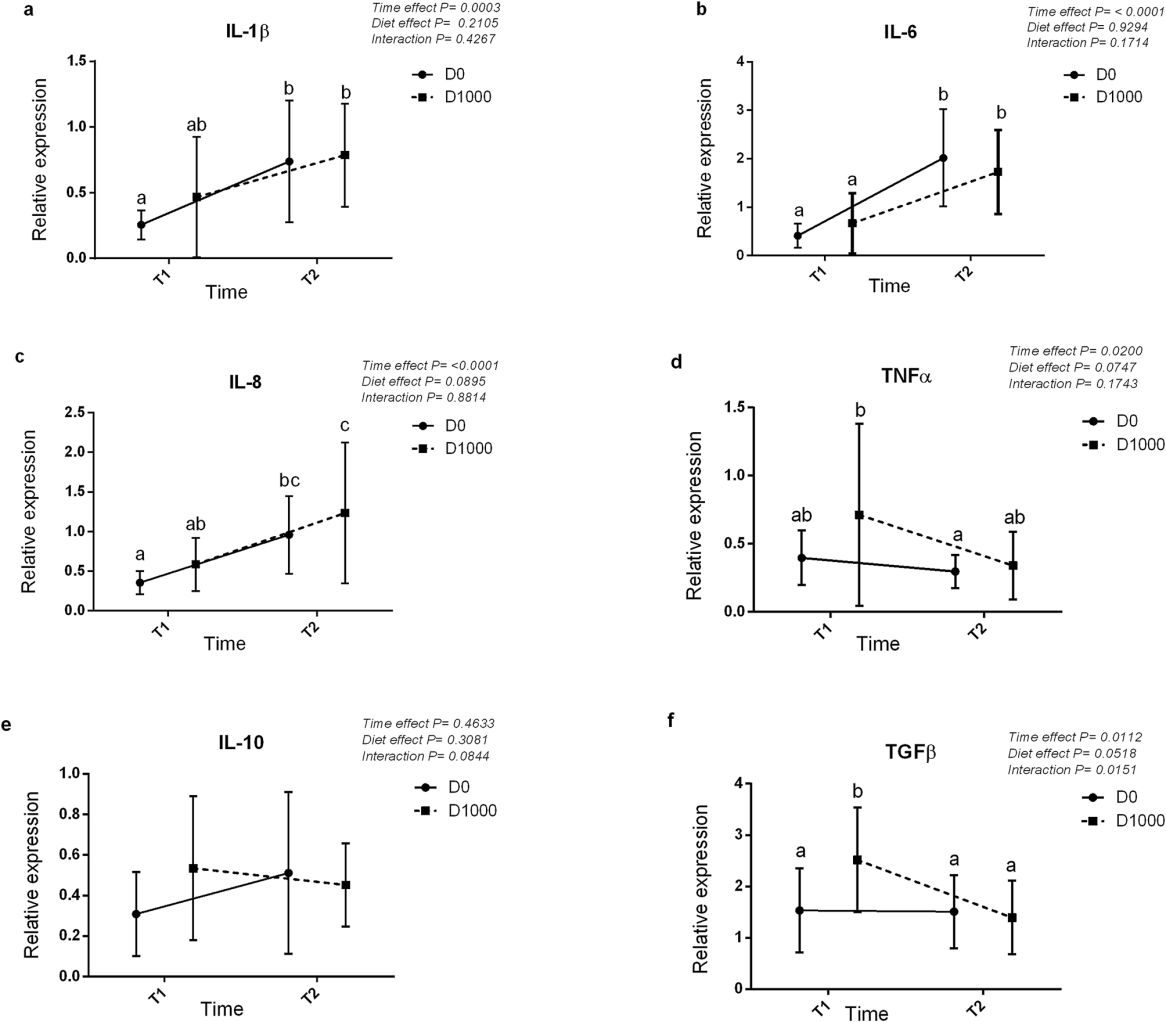
Two-way ANOVA analysis showing the expression of six cytokines genes (**a**–**f**) involved in the immune and inflammatory response in intestine among fish fed with D0 and D1000 diets at T1 (day 71) and T2 (after suboptimal rearing conditions).

The gene expression of six genes involved in the immune and inflammatory response are presented in Figs. 1 and 2. The comparison of gene expression in the intestine of fish fed different diets and sampled at the end of the feeding trial showed the upregulation of certain analysed target genes among different treatments (Fig. 1). Particularly, a significant increase in the gene expression of IL-8 (*P* = 0.0288) (Fig. 1c), one of the pro-inflammatory analysed cytokines, and IL-10 (*P* = 0.035) (Fig. 1e) and TGFβ (*P* = 0.000313) (Fig. 1f), the analysed anti-inflammatory cytokines, was observed at increasing levels of OA and NIC inclusion. The comparison of gene expression in the intestine between D0 and D1000 groups on the final day of feeding trial (named T1) and after the exposure to suboptimal rearing conditions (named T2) showed a time-dependent significant effect for five out of six cytokine genes analysed (Fig. 2). Particularly, in D0 group, IL-1β, IL-6 and IL-8 genes (Fig. 2a–c respectively) were significantly upregulated after exposure to suboptimal rearing conditions (T2), while in D1000 group at T2, IL-6, IL-8 genes (Fig. 2b,c respectively) were significantly upregulated, and the TGFβ (Fig. 2f) gene was significantly downregulated.

### Sea bass gut microbiota composition and biodiversity during the feeding trial

**Figure 3:**
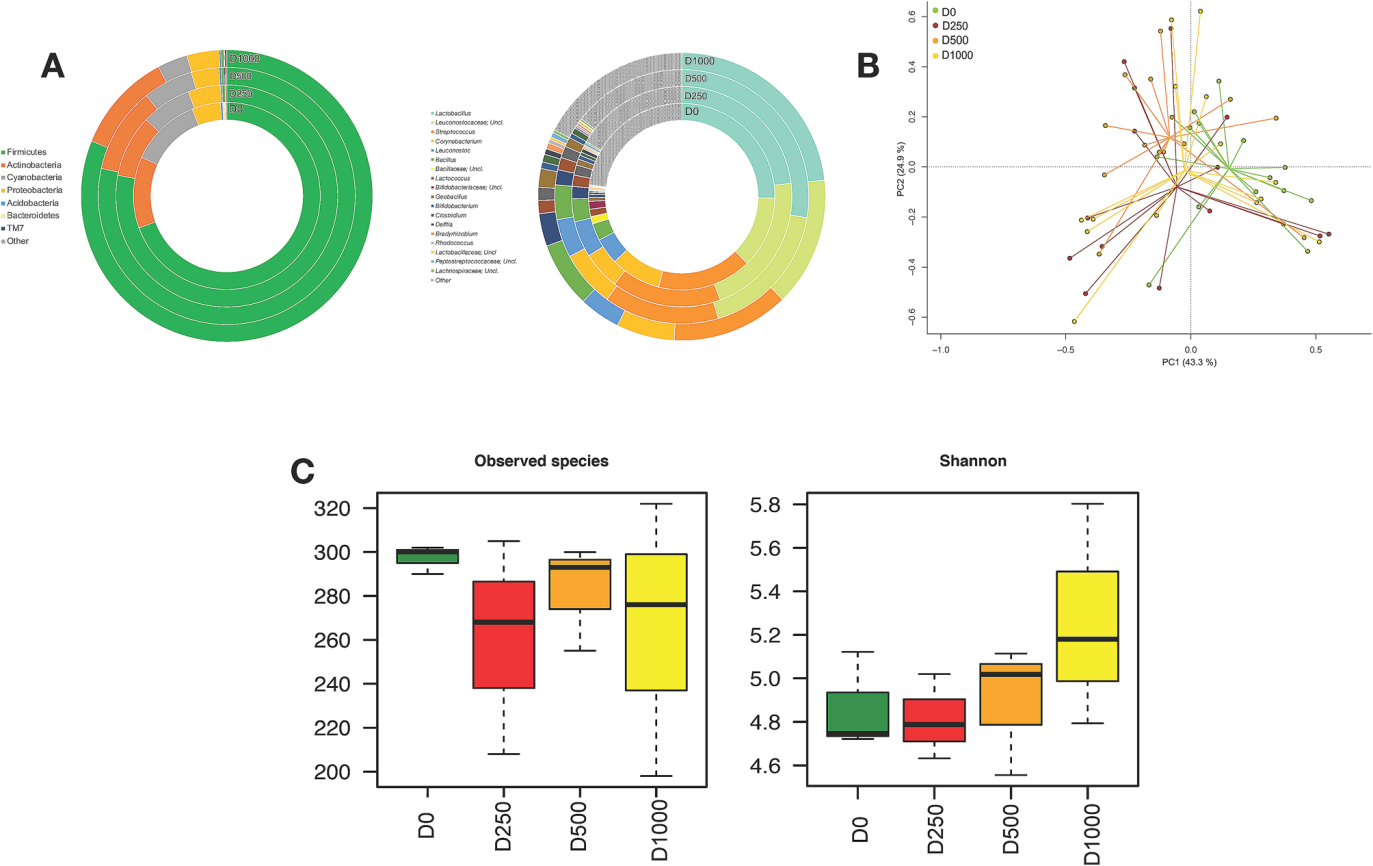
Autochthonous intestinal microbiota from sea bass *Dicentrarchus labrax* fed with control diet (D0) and different doses of microencapsulated OA and NIC (D250, D500 and D1000). (**A**) Average phylogenetic profiles of the gut microbiome of sea bass are provided at phylum (left) and genus (right) level; Phyla and genus which represent < 1.5% of the autochthonous community were included in ‘Others’. (**B**) Principal Component Analysis (PCA) of the Euclidean distance of sea bass gut microbial ecosystem after the assumption of different diets (D0, D250, D500 and D1000). A significant separation among groups was observed [P value = 0.03; Permutation test with pseudo-F ratios (Adonis)]. (**C**) Alpha diversity of the sea bass GM for all diet assumption was estimated with observed species and Shannon metrics.

The taxonomic characterisation of the sea bass gut microbial community was performed by using the 16S rRNA gene sequencing approach. The first analysis was carried out to compare the gut microbiota (GM) composition between controls (D0) and fish treated with a different dose of microencapsulated OA and NIC (D250, D500 and D1000). At phylum level, the most represented taxa were Firmicutes, Actinobacteria and Cyanobacteria (Supplementary Table S1). At genus levels, however, a dominance was identified of the genera assigned to *Lactobacillus*, and an unclassified genus belonging to *Leuconostocaceae* and *Streptococcus* families*,* (Fig. 3A) (Supplementary Table S1). In order to explore the variation of the overall GM compositional structure in sea bass after different diet treatments a Principal Component Analysis (PCA) based on the Euclidean distances in the whole sample set was performed. The PCA showed a significant separation among controls and the three different treatments in the bi-dimensional space, [*P* = 0.03; Permutation test with pseudo-F ratios (Adonis)] (Fig. 3B). In addition, different metrics were used to estimate microbial α-diversity, including Shannon and observed species (Fig. 3C). Despite the lack of statistical significance, both metrics highlighted the reduction of the sea bass gut microbial ecosystem diversity from D0 to D500, followed by an increase of the biodiversity for the D1000 diet (Fig. 3C).

**Figure 4:**
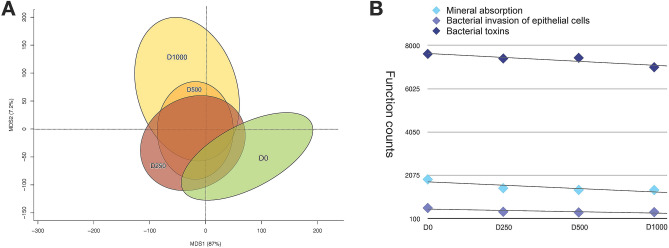
Profile of putative functions encoded by the sea bass gut microbial community during the diet intervention (D0, D250, D500, D1000). Principal Component Analysis (PCA) based on Euclidean distance between samples is reported in (**A**). A significant separation among groups was observed [P value = 0.04—Permutation test with pseudo-F ratios (Adonis)]. (**B**) D1000 promotes a significant decrease in several inflammation-promoting and homeostatic functions, such as bacterial invasion of epithelial cells (*P* = 0.01—Wilcoxon rank-sum test), bacterial toxins (*P* = 0.01—Wilcoxon rank-sum test) and mineral absorption (*P* = 0.02—Wilcoxon rank-sum test).

After the first taxonomic characterisation, a functional prediction of the sea bass gut microbial ecosystem was performed by using Picrust software^42,43^. PCA based on Euclidean distances showed a significant separation among the 4 treatment groups in relation to the gut microbiome putative functions, indicating also a potential functional reconfiguration of the sea bass GM metabolic potential [*P* = 0.04—Permutation test with pseudo-F ratios (Adonis)] (Fig. 4A). In particular, different bacterial functions with a significant decrease in D1000 diet were identified, such as bacterial invasion of epithelial cells (588 counts in D0; 399 counts ± 14 S.D. in D1000; *P* = 0.01—Wilcoxon rank-sum test), bacterial toxins (7610 counts in D0; 7278 counts ± 239 S.D. in D1000; *P* = 0.01—Wilcoxon rank-sum test) and mineral absorption (1891 counts in D0; 1435 counts ± 46 S.D. in D1000; *P* = 0.02—Wilcoxon rank-sum test) (Fig. 4B).

### Diet impact on sea bass GM following suboptimal rearing conditions

**Figure 5:**
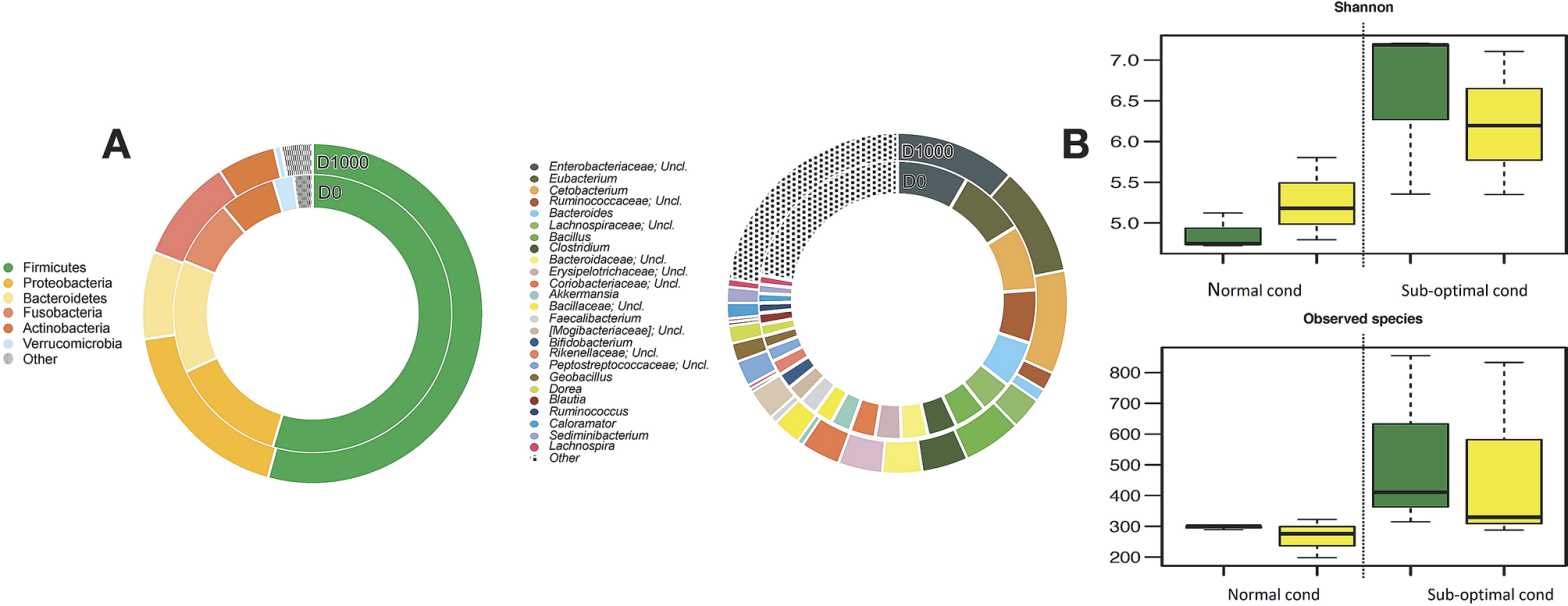
Intestinal microbiota from sea bass *Dicentrarchus labrax* fed with D0 and D1000 diets before and after the suboptimal rearing condition. (**A**) Average phylogenetic profiles of the gut microbiome of sea bass are provided at phylum (left) and genus (right) level; Phyla and genus which represent < 1.5% of the autochthonous community were included in ‘Others’. (**B**) Alpha diversity of the sea bass GM for all diet assumption was estimated with observed species and Shannon metrics (yellow circle: supplemented, green circle: control). After the suboptimal rearing conditions, the GM of sea bass in both D0 and D1000 is characterized by a significant increase of diversity compared to normal conditions (*P* = 0.01; Wilcoxon rank-sum test).

To evaluate the effect of following suboptimal rearing conditions on the GM between D0 and D1000 diets, a taxonomic analysis of the gut microbial ecosystem pre- and post- suboptimal rearing conditions was performed. In particular, the intestinal microbial community of post suboptimal rearing conditions in sea bass at phylum level, was dominated by Firmicutes, Proteobacteria, Bacteroidetes, Actinobacteria and Fusobacteria both in D0 and in D1000 treatments (Fig. 5A) (Supplementary Table S2). At the genus level, for the same sampling time the gut ecosystem was dominated by Unclassified *Enterobacteriaceae*, *Eubacterium*, *Cetobacterium*, Unclassified *Ruminococcaceae*, *Bacteroides* and *Clostridium* (Fig. 5A) (Supplementary Table S2).

In order to define the effect of suboptimal rearing condition on sea bass GM, the gut microbial diversity of sea bass pre- and post-suboptimal rearing condition was evaluated performing the α-diversity analysis by using two different metrics for both D0 and D1000 diets (e.g. Shannon and observed species) (Fig. 5B). After suboptimal rearing conditions, the gut bacterial community of sea bass showed a general increase in microbial diversity for both D0 and D1000 diets compared to the pre-suboptimal condition samples (Fig. 5B). In particular, D1000 diet was characterised by the maintenance of the GM diversity comparable to D1000 baseline (pre-suboptimal rearing condition D1000 diet).

## Discussion

Performance registered in this trial was in line with the optimal growth rate of this species, and highlighted the adequacy of the experimental design and rearing conditions applied to the fish species studied. At the end of the trial, fish tripled their weight (from 13.23 ± 0.07 g to 56.41 ± 0.42 g), and no mortality was observed. Results from the feeding trial period showed no significant differences in growth performance and feed utilisation of fish receiving D0 treatment in comparison to those fed with OA and NIC blend at different dietary inclusion levels (D250, D500, D1000).

To date, no studies have tested the effect of a dietary blend of citric acid, sorbic acid, thymol and vanillin on marine fish species. Some studies have investigated the effects of OA or botanicals, alone or in blends with other compounds, on growth performance of fish species, finding no effects on growth performance. In fact, fish studies with OA have been generally addressed to promoting fish health and disease resistance, modulation of gastro-intestinal pH and enhancement of secretion of pancreatic enzymes^24^; furthermore, studies with phytogenics have been focused on their action as immunostimulants or antimicrobials^37^.

In keeping with our observations, as examples, no significant differences in growth performance were found in European sea bass juveniles between fish fed with a diet enriched with butyrate, compared with fish fed a control diet^8^. Similarly, no beneficial growth effects of OA blends (combining formic acid, ammonium formate, propionic acid or benzoic acid, fumaric acid, and hydroxy analogue of methionine) were detected in olive flounder (*Paralichthys olivaceus*) nor in hybrid tilapia fed with potassium diformate at different inclusion levels^9,44^ or in red tilapia (*Oreochromis *sp.) fed an OA blends and potassium diformate^45^. In these studies it was supposed that the final impact of OA in aquaculture may be dependent on several biotic and abiotic factors, such as age^46^, type and level of organic acids^27,47^, diet composition^16,48^, culture conditions^13^ and species digestive physiology. In the present study, differently from the aforementioned ones, the active ingredients OA + NIC were microencapsulated in a lipid matrix that allows their release once lipases start to be secreted in the intestinal tract^49^ so hypothetically there is not an effect mediated by acidification in the stomach.

Despite the lack of significant increase in performance at the tested inclusions, it was possible to determine positive effects in the modulation of GM as well as a boosting of anti-inflammatory functions. Although in the literature only a few studies on the effect of OA or phytogenics on host health of aquaculture species are available, they demonstrate the beneficial effects of including OA or botanicals on disease resistance in both finfish and shellfish^9,45,50,51^. In particular, an increase in disease resistance to several fish pathogens such as *Edwarsiella tarda*,* Vibrio anguillarum*,* Aeromonas hydrophila* and *Streptococcus agalactiae* was demonstrated for fish fed diets with the inclusion of OA^13,14,31,45,52^. These beneficial effects can be due to the antimicrobial mode of action which relies on altering bacterial cell permeability/fluidity and quorum sensing systems. Also, other recognised effects are related to the reduction in gut oxidative stress, stabilisation of intestinal microbiota, and modulation of the immune system via scavenging of free radicals^53^.

Among immune parameters, cytokines play an important role and have been commonly used as the reference genes in studies of immune regulation^54–56^. Inflammatory cytokines are classified as pro-inflammatory and anti-inflammatory cytokines. Pro-inflammatory cytokines includes IL-1β, IL-6, IL-8 and TNF-α and are upregulated during infective and inflaming reactions^56,57^. Instead, anti-inflammatory cytokines such as IL-10 and TGF-β are downregulated during the inflammatory process^58,59^. However, only a few studies have investigated cytokine gene expression level induced in the intestine by OA or NIC inclusions^8,41,54^. In our study, three out of six investigated target genes related to inflammatory response and reinforcement of epithelial defence showed a dose-dependent upregulation; IL-8, IL-10 and TGF-β showed significantly higher expression values at increasing levels of OA and NIC inclusion. A previous study in European sea bass showed a significant upregulation of IL-10 after butyrate treatment, but not of IL-8^8^. A significant increase of the mRNA expression levels of anti-inflammatory cytokines (IL-10 and TGF-β) was also induced in carp by dietary supplementation with conjugated linoleic acid^59^. Moreover, citric acid inclusion was able to upregulate gene expression of the anti-inflammatory cytokine TGF-β in fish fed soybean meal protein diets^57^. For anti-inflammatory cytokines, including IL-10 and TGF-β, a protective effect on epithelial integrity was also described, decreasing intestinal epithelial permeability by raising transepithelial electrical resistance^58,60^. In this respect, the OA and NIC blend tested showed a beneficial effect enhancing anti-inflammatory response at intestinal level and thus protecting epithelial integrity. The exposure of fish to suboptimal rearing conditions induced a time-dependent significant effect for five out of six cytokine genes analysed, with an upregulation of pro-inflammatory cytokines including IL-1β, IL-6 and IL-8, and a downregulation of the anti-inflammatory cytokine TGF-β showing that suboptimal rearing conditions can induce an inflammatory process at intestinal level. The blend of OA + NIC was able to upregulate anti-inflammatory cytokines before and after the exposure to these suboptimal rearing conditions, showing the potential immune-boosting action of this microencapsulated feed additive.

The GM plays a critical role on host health as a defensive barrier and on metabolism under different feeding and environmental condition in marine fish species^61,62^. To the best of our knowledge, this study represents the first investigation on the effects of a blend of citric acid, sorbic acids, vanillin and thymol on the gut bacterial community of marine fish species. In addition, the encapsulation which is the peculiarity of this blend, suggests that the volatile compounds (NIC) are protected from degradation in the stomach^34^ and thus arrive intact in the intestine where they can exert their effects on GM. According to our findings, the gut bacterial community is dominated by Firmicutes followed by Actinobacteria, Cyanobacteria and Protebacteria, regardless of the type of diet. At genus level the gut bacterial community was mainly represented by the lactic acid bacteria (LAB) *Lactobacillus, Leuconostocaceae* and *Streptococcus* covering over 50% of the total relative bacterial abundance. Our data are in line with previous GM characterisation of the distal intestinal content of sea bass reared with different FM dietary level^63^. In that study, the genera belonging to LAB mainly *Lactobacillus, Leuconostoc, Lactococcus, Streptococcus* were the most represented in all the treatments covering over 40% of the relative abundances. LAB are considered to be the most promising bacterial genera as probiotics in aquaculture due to their ability to stimulate host gastrointestinal development, digestive function, mucosal tolerance and immune response, and to improve disease resistance, even if the underlying mechanism is still poorly understood^64^. The results of the present study reinforce previous observation that the dominance of LAB in the intestinal lumen of sea bass is associated with optimal health condition and growth^63,65,66^. No significant effects of the blend of OA and NIC on any specific component at phylum and genus level were detected (Wilcoxon ran-sum test, *P* > 0.05, FDR correction). However, an overall impact on GM has been observed, as highlighted by the PCA based on Euclidean distance which showed a significant separation among the treatments. In particular a moderate increase (*P* = 0.08) was detected for the LAB *Lactobacillus and Leuconostocaceae* under D500 and *Leuconostoc* in D250 (*P* = 0.12). OA in fish species can exert their antimicrobial activity on microbes by directly lowering the pH of the environment via releasing H^+^ ions and thus preventing and/or impeding the growth and proliferation of acid-sensitive bacteria^1^. Previous studies on Nile tilapia and red hybrid tilapia using standard culture method techniques have shown a reduction in total faecal bacteria counts following increasing dietary inclusion of malic acid (0–10 g kg diet^−1^), potassium diformate (2 g kg diet), or a blend of OA (1–3 g kg diet^−1^)^45,67^. Using a similar technique, dietary Na-butyrate supplementation increased the abundance of *Lactobacillus* in the intestine of grass carp while dietary inclusion of potassium diformate promoted the colonisation of beneficial LAB in Nile tilapia^14,68^. According to Hassaan et al*.*^67^ explaining the phenomenon of qualitative and quantitative changes in the ecosystem of fish intestinal microbiota following OA dietary administration requires further clarification and more extensive microbiological analysis. Among the few studies available in literature conducted using next generation sequencing (NGS) on this topic, Rimoldi et al*.*^69^ evaluated the dietary effect of OA blend including propionic, butyric, caproic, heptanoic, caprylic and lauric on GM in sea bream. Although the authors could not discriminate significant differences in the abundances of specific taxa using FDR methods, the relative abundance of lactic acid bacteria belonging to the *Leuconostocaceae* and *Lactobacillaceae* families was positively affected by dietary OA. In addition, in agreement with our findings the authors also detected a reduction in the alpha-diversity indices following the inclusion of OA supplementation, which is also in line with the reduced faecal bacterial counts previously mentioned. It has been postulated that small doses of OA may exert prebiotic properties, stimulating the development of non-pathogenic, acidophilic and saprophytic bacteria, whereas at higher doses it can exhibit bacteriostatic and bactericidal properties. In the present study, this was observed for *Leuconostocaceae* which reached their maximum abundance in D250, while *Lactobacillus* abundance was higher under D500. It should also be mentioned that the bacteria belonging to the genus *Bacillus* sp., even if not significant*,* increased from 3.0 to 7.4% at increasing OA and NIC. It has been observed that *Bacillus *sp. may positively contribute to nutrition, can inhibit pathogen adherence and colonisation, may affect the immune system decreasing inflammation via the up-regulated secretion of anti-inflammatory cytokines, and may have potential as probiotics in fish^70^ and fish larvae^71^. In agreement with the present study the oral administration of the OA poly-β-hydroxybutyrate (PHB) have been found to increase *Bacillus* sp. in gibel carp (*Carassius gibelio*)^72^ and soiny mullet (*Liza haematocheila*)^73^. The authors suggested that dietary PHB supplementation changed the intestinal microbiota structure, increased probiotics growth and abundance, and inhibited opportunistic bacterial pathogen growth. Following the first taxonomic evaluation, PICRUSt was applied to predict metagenomic functional content from previously-obtained 16S marker gene survey^42,43^.

PCA based on Euclidean distance showed a significant separation among the 4 treatment groups based on the putative functions encoded in the microbiome, indicating a functional reconfiguration of the GM metabolic potential. In particular, the blend of OA and NIC promotes a significant decrease in several inflammation-promoting and homeostatic functions, such as bacterial invasion of epithelial cells and bacterial toxins.

Positive impacts like enhancement on immunity could be associated with the increase of bacteria belonging to Firmicutes such as *Lactobacillus Leuconostoc*, and *Bacillus* sp. In this regard, LAB could have an active role in host defence against pathogenic bacterial invasion at the intestinal level. It is known that lactic acid bacteria inhibit the growth of pathogens by producing antibacterial compounds, such as lactic acid, hydrogen peroxide and bacteriocins, and by releasing biosurfactants^69^ .

Suboptimal rearing conditions (high temperature and low oxygen), affected gut bacterial community relative abundance. The observed decrease of Firmicutes was mainly associated with the decrease of LAB relative abundance such as *Lactobacillus* (pre-post 24.2 ± 1.5% 0.6 ± 0.2%, respectively), *Leuconostocaceae* (pre-post 13.4 ± 1.4% 0.1 ± 0.0%, respectively) and *Streptococcus* (pre-post 14.8 ± 2.2% 0.5 ± 0.2%, respectively). Interestingly, among potential beneficial taxa *Bacillus* sp. was not affected by suboptimal rearing conditions (pre-post 5.2 ± 3.1% 4.6 ± 1.2%, respectively), which is consistent with its ability to live in extreme environmental conditions such as high pH^74^, high temperature^75^ and high salinity^76^.

Few studies reported GM interaction with unfavourable environmental conditions in fish species^41,64,77^ and no data in this regard are available for European sea bass.

In farmed Tasmanian Atlantic salmon, high water temperature influenced the function and diversity of GM, revealing a decrease in LAB abundance during warmer months^78^.

According to Zarkasi et al*.*^79^, seasonal changes in temperature is one of the influencing factors which may affect the GM structure. The authors revealed that anaerobe classes such as Clostridia, Bacteroidia and Fusobacteria peaked in the summer months and then declined in winter, while, in most cases, facultative anaerobes like LAB showed an inverse trend. Moreover, variations in gut microbial community abundance could be not only affected by seasonal factors but also related to fish physiology and diet^79,80^. In addition, in the present study the reduction of the oxygen level in the environment may have contributed to the modulation of gut microbiota favouring the presence of obligate anaerobic Firmicutes such as Clostridiales. On the other hand, after suboptimal rearing conditions, Proteobacteria showed a relative increase in abundance (pre-post 5.5 ± 0.7% 15.9 ± 3.4%, respectively), mainly represented by the family of *Enterobacteriacee* (pre-post 0.1 ± 0.04% 9.9 ± 2.2%, respectively). The increase of Proteobacteria is often associated with environmental stress: high temperature, poor water quality, and high organic content^79^. For this reason, *Enterbacteriaceae* family is often considered as an indicator of environmental pollution, and has been reported as opportunistic pathogens which contribute to the onset and severity of bacterial infections in fish^79,81,82^. Focusing on the effect of dietary AO and NIC after the suboptimal rearing conditions, despite a general increase in gut bacterial community α-diversity, fish fed the OA and NIC blend (inclusion level D1000) tend to maintain GM diversity comparable to the pre-suboptimal conditions.

In conclusion, the different inclusion level of OA and NIC blend in diets did not promote growth, feed utilisation and feed intake in European sea bass; but higher inclusions need to be further tested. However, results of cytokines gene expression indicate that dietary OA and NIC blend seems to enhance a beneficial effect on anti-inflammatory response at intestinal level, thus protecting epithelial integrity. Dietary OA and NIC seems to exert prebiotic properties stimulating the development of beneficial bacteria taxa such as *Lactobacillus*,* Leuconostoc*, and *Bacillus* sp. and seems to induce a potential functional reconfiguration of GM, promoting a significant decrease in several inflammation-promoting and homeostatic functions. For the first time in this species we observed that exposure to suboptimal rearing conditions (high temperature and low oxygen) is able to modify gut bacterial community structure while reducing LAB and increasing Proteobacteria (mainly belonging to *Entebacteriaceae* family); findings which are consistent with the significant upregulation of five out of the six analyzed cytokine genes observed at this time. Despite the general increase in the gut bacterial community α-biodiversity observed after the suboptimal condition, the microencapsulated blend OA + NIC studied seems to mitigate this effect maintaining a level comparable to the normal rearing condition.

## Materials and methods

### Ethical statement

The experiment was designed according to the guidelines of the current European Directive (2010/63/EU) on the protection of animals used for scientific purposes. The experimental protocol was approved by the Ethical Committee of the University of Bologna (Italy) (protocol N°610).

### Experimental diets

A commercial diet (VITA 1.5, Veronesi, A.I.A SpA, Verona, Italy, pellet diameter 1.6–1.9 mm) was coated with increasing dose (D) levels (0, 250, 500, 1000 ppm; D0, D250, D500 and D1000, respectively) of a blend of OA and NIC providing 25% citric acid, 16.7% sorbic acid, , 1.7% thymol and 1% vanillin in a matrix of hydrogenated fats (Aviplus Aqua -Vetagro SpA, Reggio Emilia, Italy; US patent # 7,258,880; EU patent # 1-391-155B1; CA patent # 2,433,484). The blend was chosen according to previous study in Pacific white shrimp and rainbow trout^40,41^. Commercial diet was formulated including practical ingredients for sea bass (fishmeal, wheat gluten, soy protein concentrate, fish oil, wheat, soybean oil and soybean meal) to contain 49.8% protein; 20.6% lipid; 7.3% ash; 5.6% moisture and with an energy content of 5152.6 cal/g. Vitamins and mineral premix was included fulfilling recommendations for marine fish species given by NRC (2011)^83^.

### Fish and feeding trial

The experiment was carried out at the Laboratory of Aquaculture, Department of Veterinary Medical Sciences of the University of Bologna, Cesenatico, Italy. European sea bass juveniles were obtained from an Italian hatchery. At the beginning of the trial 60 fish (13.23 ± 0.18 g) per tank were randomly distributed into twelve 500 L square flat bottom. Each diet was administered to triplicate groups. Tanks were provided with natural seawater and connected to a closed recirculating system (overall water volume: 7000 L; Oxygen level 8.0 ± 1.0 mg L^−1^; Temperature 23 ± 1.0 °C, Salinity 25 g L^−1^ ) according to Parma et al.^50^. The feeding trial lasted 71 days. Feed was provided by hand to visual satiation twice a day (8.30 and 16.00) for 6 days a week, while one meal was supplied on Sundays.

### Suboptimal rearing conditions

In order to explore the effect of OA and NIC during unfavourable Mediterranean summer conditions, after the end of the trial, fish belonging to D0 and D1000, were maintained at high temperature (30.0 ± 0.4 °C) and low oxygen (4.6 ± 0.6 mg L^−1^, 67.6 ± 5.0% saturation level) for 8 days while keeping the same feeding conditions. Specifically, temperature was increased at a rate of 2 degree day^−1^ while oxygen concentration was decreased from 8.0 ± 1.0 mg L^−1^, 105.0 ± 1.0% to 4.6 ± 0.6 mg L^−1^, 67.6 ± 5.0% within 24 h. Temperature and oxygen level were constantly monitored through an automatic system regulated by a software programme (B&G Sinergia snc, Chioggia, Italy). The environmental parameters were chosen according to previous studies on oxygen and thermal tolerance of the species^84,85^. Fish were daily monitored to check any sign of mortality.

### Sampling

At the beginning, at the intermediate period (day 37) and at the end of the feeding trial, fish were anaesthetised and individually weighed. Specific growth rate (SGR), feed intake (FI) and feed conversion ratio (FCR) were calculated. Proximate carcass composition was determined at the beginning of the trial on a pooled sample of 15 fish and on pooled samples of 5 fish tank^−1^ at the end of the trial. Protein efficiency ratio (PER), gross protein efficiency (GPE), lipid efficiency ratio (LER), and gross lipid efficiency (GLE) were calculated. At the end of the trial, viscerosomatic index (VSI), hepatosomatic index (HSI) and condition factor (CF) were also obtained from 5 fish per tank^41^.

Concerning immunological parameters, at the beginning (15 fish in total), at the end of the feeding trial (5 animals tank^−1^) and at the end of the suboptimal rearing conditions (5 animals tank^−1^) fish were sampled to assess inflammatory and immune response gene expression of distal intestinal mucosa^41^.

At the end of the feeding trial, and at the end of suboptimal rearing conditions, samples of distal intestine content from 5 fish tank^−1^ were also individually collected and placed at − 80 °C for gut bacterial community characterisation^41,63^. After the suboptimal rearing conditions, due to the moderate amount of individual gut content collected during this period, the analyses were performed on a pool collected from 5 fish per tank according to Parma et al*.*^53,65^.

### Gene expression analyses by real-time polymerase chain reaction

Total RNA was isolated from 50 mg of intestine samples stored in RNA Later (Sigma) using the NucleoSpin RNA extraction kit following the manufacturer’s instructions. The RNA extraction protocol includes a treatment with DNAse I in order to remove genomic DNA. The first strand of cDNA was synthesised by reverse transcription using the GoScript Reverse Transcriptase (Promega). cDNA concentration was quantified using a Qubit Fluorometer (ThermoFisher). Real-time PCR was performed with an ABI PRISM 7300 instrument (Applied Biosystems) using BRYT Green GoTaq qPCR (Promega). 10 ng of each cDNA sample was added to a reaction mix containing 2 × GoTaq qPCR Master Mix (Promega), 300 nM of CXR and 200 nM of each primer. The primers used for Interleukin 1β (IL-1β), 6 (IL-6), 8 (IL-8), 10 (IL-10), Tumor necrosis factor α (TNF-α), Transforming growth factor β (TGF-β), are shown in Table 3. Reaction mixtures were incubated for 2 min at 95 °C, followed by 50 cycles of 10 s at 95 °C, 30 s at 60 °C, and finally 15 s at 95 °C, 1 min 60 °C and 15 s at 95 °C (dissociation stage). Before the experiments, the specificity of each primer pair was studied using positive and negative samples. A melting curve analysis of the amplified products validated the primers for specificity. After these verifications, all cDNA samples were analysed in triplicate. Negative controls with no template were always included in the reactions. For each sample, gene expression was normalised against beta-actin (β-actin) gene and expressed as 2-ΔΔCt, where ΔCt is determined by subtracting the β-actin Ct value from the target Ct. Gene expression of untreated and treated samples collected at time one and two were expressed as “fold changes” relative to untreated controls sampled at time zero^41^.

**Table 3:** Primer sequences used for gene expression analyses of immune genes.

Gene	Abbreviation	GenBank ID	Primer sequence (5′–3′)	Amplicon (bp)	References
β-actin	β-act	AJ493428	ACCCAGTCCTGCTCACAGAG CGGAGTCCATGACAATACCAGTG	165	This study
Interleukin 1 beta	IL-1β	AJ311925	ATCTGGAGGTGGTGGACAAA AGGGTGCTGATGTTCAAACC	106	Sepulcre et al.^93^
Interleukin 6	IL-6	AM490062	ACTTCCAAAACATGCCCTGA CCGCTGGTCAGTCTAAGGAG	170	Sepulcre et al.^93^
Interleukin 8	IL-8	AM490063	GTCTGAGAAGCCTGGGAGTG GCAATGGGAGTTAGCAGGAA	110	Sepulcre et al.^93^
Interleukin 10	IL-10	DQ821114	CGACCAGCTCAAGAGTGATG AGAGGCTGCATGGTTTCTGT	199	Sepulcre et al.^93^
Tumor necrosis factor alfa	TNF-α	DQ200910	AGCCACAGGATCTGGAGCTA GTCCGCTTCTGTAGCTGTCC	112	Sepulcre et al.^93^
Transforming growth factor beta	TGF-β	AM421619	GACCTGGGATGGAAGTGGAT CAGCTGCTCCACCTTGTGTTG	225	Faliex et al.^94^

### Gut bacterial community DNA extraction and sequencing

Total microbial DNA was extracted and analysed from individual distal intestine content obtained from 5 fish per tank as previously reported in Parma et al*.*^65^ Afterwards, to perform the 16S rRNA gene analysis, the V3–V4 hypervariable region was amplified using the 2 × KAPA HiFi HotStart ReadyMix (KAPA Biosystems) with the addition of 341F and 785R primers^86^ modified with Illumina adapter overhang sequences. Briefly, the thermal cycle consisted of an initial denaturation at 95 °C for 3 min, 30 cycles of denaturation at 95 °C for 30 s, annealing at 55 °C for 30 s and extension at 72 °C for 30 s, and a final extension step at 72 °C for 5 min. As recommended in the Illumina protocol “16S Metagenomic Sequencing Library Preparation” for the MiSeq system, PCR reaction were cleaned up by using Agencourt AMPure XP magnetic beads. A limited-cycle PCR was performed to obtain the indexed library using Nextera technology, followed by a second AMPure XP magnetic beads clean-up step. Sequencing was performed on Illumina MiSeq platform using a 2 × 250 bp paired-end protocol according to the manufacturer’s instructions (Illumina, San Diego, CA). The sequencing process resulted in a total of 1,857,956 high quality reads and processed by using a combined pipeline of PANDAseq^87^ and QIIME^88^ .Quality-filtered sequences were clustered into OTUs at 97% similarity threshold using UCLUST^89^ and taxonomy was assigned using the RDP classifier and the Greengenes database (May 2013 release). α-diversity was evaluated using two different metrics: Shannon and observed species. Euclidean distance was used to perform Principal Component Analysis (PCA). Predictive functional trait imputations for the microbial community was performed using the PICRUSt software^42,43^ PCA and bar plots were built using the R packages “Made4”^90^ and “Vegan” (https://cran.r-project.org/web/packages/vegan/index.html).

### Analytical methods

Diets and whole body were analysed for proximate composition. Moisture content was measured by weight loss after drying samples in an oven at 105 °C until a constant weight was achieved. Total lipids were analysed according to Bligh and Dyer's^91^ extraction method. Crude protein was determined as total nitrogen (N) through the Kjeldahl method and multiplying N by 6.25. Ash content was evaluated by incineration to a constant weight in a muffle oven at 450 °C. Gross energy was measured by a calorimetric bomb (Adiabatic Calorimetric Bomb Parr 1261; PARR Instrument, IL, U.S.A)^92^.

### Statistical analysis

All data are presented as mean ± standard deviation (SD). Results on growth, proximate composition, nutritional indices, biometric indices, and cytokine expression at the end of the feeding trial were analysed by linear regression model in order to measure the effect of the increasing doses of OA blend on considered data.

Data of cytokine expression measured before and after the suboptimal rearing conditions period on fish treated with D0 and D1000, were analysed by a two-way ANOVA considering diet and time as independent factors, and in case of significance (P value ≤ 0.05) Tukey’s post hoc test was performed. The normality and/or homogeneity of variance assumptions were validated for all data preceding ANOVA test.

The R packages “Stats” and “Vegan” were used to perform gut microbiota statistical analysis. In particular, to compare the microbiota structure among different groups for alpha and beta-diversity, a Wilcoxon rank-sum test was used while data separation in the PCA was tested using a permutation test with pseudo-F ratios (function “Adonis” in the “Vegan” package)^66^. For GM analyses, *P* values were appropriately corrected for multiple comparisons using the Benjamini–Hochberg method. False discovery rate (FDR) ≤ 0.05 was considered as statistically significant. Data were analysed using GraphPad Prism 6.0 for Windows (Graph Pad Software, San Diego, CA, USA) and Rstudio interface for R (https://www.r-project.org).

## Supplementary information

Supplementary information.
