# Supplementary material for: Effects of dietary organic acids and nature identical compounds on growth, immune parameters and gut microbiota of European sea bass
Source: Sci Rep. 2020 Dec 7;10:21321. doi: 10.1038/s41598-020-78441-9 (PMC7721706; doi:10.1038/s41598-020-78441-9)
Supplement: Supplementary file 1 — Supplementary information. [file 41598_2020_78441_MOESM1_ESM.pdf]

Effects of dietary organic acids and nature identical compounds on growth, immune parameters and gut microbiota of European sea bass

Serena Busti[1], Barbara Rossi[2], Enrico Volpe[1], Sara Ciulli[1], Andrea Piva[1,2], Federica D'Amico[3], Matteo Soverini[3], Marco Candela[3], Pier Paolo Gatta[1], Alessio Bonaldo[1*], Ester Grilli[1], Luca Parma[1]

[1]Department of Veterinary Medical Sciences, University of Bologna, Via Tolara di Sopra 50, 40064 Ozzano Emilia, Bologna, Italy;

[2]Vetagro S.p.A., Via Porro 2, 42124, Reggio Emilia, Italy;

[3]Unit of Microbial Ecology of Health, Department of Pharmacy and Biotechnology, University of Bologna, Via Belmeloro 6, 40126 Bologna, Italy

*Corresponding author: alessio.bonaldo@unibo.it

SUPPLEMENTARY INFORMATION

Supplementary Table S1. Mean relative abundance (%) ± SD (n=3) of bacterial phyla and genera detected in the hindgut content of European sea bass fed different diets at 71 days (pre-suboptimal rearing condition), and at 81 days (post-suboptimal rearing condition). D0= 0 mg Kg $^{-1}$ organic acids and and nature-identical compounds (OA, NIC) ; D250 = 250 mg Kg $^{-1}$OA, NIC; D500 = 500 mg Kg $^{-1}$ OA, NIC; D1000 = 1000 mg Kg $^{-1}$ OA, NIC. Only taxa with relative abundance >0.05% in at least 1 samples were included. Taxa wich represent <0.05% abundance were grouped “Other”.

	Pre- suboptimal rearing condition								Post-suboptimal rearing condition				
**Diet**	**D0**		**D250**		**D500**		**D1000**			**D0**		**D1000**	
**Phylum**	Mean	SD	Mean	SD	Mean	SD	Mean	SD		Mean	SD	Mean	SD
Firmicutes	**69.4**	3.6	**78**	10.8	**78.4**	3.9	**77.7**	4.5	*Firmicutes*	**54.6**	3.2	**54.2**	1.4
Actinobacteria	**12.3**	1.9	**9.1**	1.6	**10.8**	0.5	**10.5**	1.3	*Proteobacteria*	**13.5**	3.8	**18.4**	0.8
Cyanobacteria	**12.2**	2.8	**7.3**	8.2	**6.3**	4.6	**3.4**	1.2	*Fusobacteria*	**7.7**	6.0	**9.9**	2.2
Proteobacteria	**5**	0.8	**4.7**	1.7	**3.5**	1.4	**6**	4.0	*Bacteroidetes*	**13**	3.7	**8.3**	1.3
Acidobacteria	**0.4**	0.2	**0.2**	0.2	**0.3**	0.3	**0.8**	1.1	*Actinobacteria*	**6.5**	1.5	**5.6**	0.3
Bacteroidetes	**0.4**	0.2	**0.4**	0.3	**0.3**	0.1	**0.4**	0.3	*Cyanobacteria*	**0.3**	0.2	**0.9**	0.8
Chloroflexi	**0**	0.0	**0**	0.0	**0**	0.0	**0.4**	0.6	*Verrucomicrobia*	**2.4**	1.2	**0.6**	0.5
TM7	**0.1**	0.1	**0.1**	0.1	**0.2**	0.1	**0.3**	0.3	*Chlamydiae*	**0.1**	0.1	**0.3**	0.3
Planctomycetes	**0**	0.0	**0**	0.0	**0**	0.0	**0.1**	0.2	*Acidobacteria*	**0.1**	0.1	**0.2**	0.1
WPS-2	**0**	0.0	**0**	0.0	**0**	0.0	**0.1**	0.2	*Chloroflexi*	**0.1**	0.2	**0.1**	0.2
Verrucomicrobia	**0.1**	0.0	**0**	0.0	**0**	0.0	**0.1**	0.1	*Planctomycetes*	**0.1**	0.1	**0.1**	0.1
Other	**0.1**	0.1	**0.1**	0.0	**0.1**	0.0	**0.2**	0.2	*Other*	**1.3**	1.2	**1.2**	1.3
**Diet**	**D0**		**D250**		**D500**		**D1000**			**D0**		**D1000**	
**Genus**	Mean	SD	Mean	SD	Mean	SD	Mean	SD		Mean	SD	Mean	SD
Lactobacillus	**25.2**	0.8	**23.1**	3.4	**27.6**	1.5	**23.2**	4.6	*Enterobacteriaceae*	**8.3**	5.4	**11.5**	2.0
Leuconostocaceae	**12.4**	3.2	**21.3**	4.2	**17.7**	0.9	**14.5**	7.0	*Eubacterium*	**7.9**	5.9	**10.5**	3.3
Streptococcus	**16.3**	3.9	**16.1**	3.1	**14.7**	4.0	**13.2**	7.0	*Cetobacterium*	**7.7**	5.9	**9.8**	2.2
Streptophyta	**12.2**	2.8	**7.3**	8.2	**6.3**	4.6	**3.2**	1.6	*Bacillus*	**3.7**	2.5	**5.5**	1.7
Bacillus	**3.0**	2.4	**3.4**	1.9	**4.4**	3.2	**7.4**	6.8	*Clostridium*	**3.4**	1.2	**4.4**	0.2
Corynebacterium	**8.8**	1.2	**5.6**	1.2	**7.2**	0.5	**6.6**	0.8	*Erysipelotrichaceae*	**3.0**	1.7	**4.2**	0.9

Leuconostoc	**4.5**	0.3	**5.3**	0.6	**4.9**	1.2	**4.6**	2.6	*Bacteroidaceae*	**3.1**	2.6	**3.8**	1.3
Bacillaceae	**1.3**	0.6	**1.8**	1.0	**1.9**	0.8	**3.7**	3.5	*Coriobacteriaceae*	**3.0**	1.7	**3.8**	0.6
Geobacillus	**0.6**	0.5	**0.9**	0.4	**1.2**	0.6	**2.1**	2.4	*Lachnospiraceae*	**3.8**	0.1	**3.1**	0.0
mitochondria	**2.9**	1.2	**2.6**	2.6	**1.9**	0.9	**1.8**	0.7	*Mogibacteriaceae*	**2.0**	1.6	**3.0**	0.8
Lactococcus	**1.3**	0.2	**1.7**	0.4	**1.6**	0.3	**1.5**	0.6	*Bacillaceae*	**2.2**	1.6	**2.7**	1.0
Bifidobacteriaceae	**1.4**	0.9	**1.5**	0.3	**1.5**	0.1	**1.5**	1.0	*Peptostreptococcaceae*	**1.7**	1.1	**2.4**	0.5
Clostridium	**0.4**	0.2	**0.5**	0.5	**0.8**	0.6	**0.9**	0.7	*Ruminococcaceae*	**6.2**	3.7	**1.8**	1.1
Bifidobacterium	**0.6**	0.3	**0.6**	0.0	**0.6**	0.2	**0.7**	0.2	*Geobacillus*	**1.4**	1.0	**1.8**	0.1
Bradyrhizobium	**0.1**	0.1	**0.2**	0.1	**0.2**	0.2	**0.7**	0.9	*Dorea*	**1.4**	0.4	**1.7**	0.6
Delftia	**0.2**	0.2	**0.6**	0.4	**0.2**	0.2	**0.6**	0.6	*Sediminibacterium*	**1.1**	0.9	**1.5**	0.7
Peptostreptococcaceae	**0.2**	0.1	**0.3**	0.4	**0.1**	0.1	**0.5**	0.6	*Caloramator*	**1.1**	0.8	**1.4**	0.3
Caulobacteraceae	**0.1**	0.0	**0.1**	0.1	**0.1**	0.1	**0.5**	0.7	*Carnobacterium*	**0.9**	0.8	**1.3**	0.8
Faecalibacterium	**0.1**	0.1	**0.0**	0.1	**0.1**	0.1	**0.4**	0.5	*Clostridiales*	**1.5**	1.0	**1.2**	0.9
Sphingobium	**0.0**	0.0	**0.2**	0.2	**0.2**	0.1	**0.4**	0.6	*Bacteroides*	**5.4**	3.8	**1.2**	0.9
Rhodococcus	**0.2**	0.1	**0.2**	0.1	**0.3**	0.1	**0.4**	0.2	*Delftia*	**0.6**	0.5	**1.0**	0.6
Lactobacillales	**0.6**	0.1	**0.4**	0.0	**0.3**	0.1	**0.4**	0.3	*Sphingobium*	**0.7**	0.6	**1.0**	0.5
Sporanaerobacter	**0.2**	0.1	**0.2**	0.1	**0.2**	0.2	**0.4**	0.4	*Anaerobacillus*	**0.6**	0.6	**0.8**	0.3
Blautia	**0.2**	0.2	**0.1**	0.1	**0.1**	0.1	**0.4**	0.4	*Lachnospira*	**1.0**	0.8	**0.8**	1.0
Solibacterales	**0.2**	0.1	**0.1**	0.1	**0.2**	0.2	**0.4**	0.5	*Streptophyta*	**0.2**	0.1	**0.7**	0.8
Sphingomonas	**0.1**	0.1	**0.2**	0.2	**0.1**	0.1	**0.4**	0.5	*Faecalibacterium*	**2.1**	2.6	**0.7**	0.5
Ellin6513	**0.1**	0.1	**0.1**	0.1	**0.1**	0.0	**0.4**	0.6	*Caulobacteraceae*	**0.5**	0.4	**0.6**	0.2
Ruminococcus	**0.1**	0.0	**0.2**	0.1	**0.1**	0.0	**0.4**	0.0	*Akkermansia*	**2.3**	1.2	**0.6**	0.5
Lachnospiraceae	**0.2**	0.1	**0.2**	0.2	**0.2**	0.1	**0.3**	0.3	*Methylobacterium*	**0.4**	0.3	**0.5**	0.3
Lactobacillaceae	**0.3**	0.1	**0.2**	0.1	**0.3**	0.2	**0.3**	0.3	*Eubacteriaceae;Other*	**0.4**	0.2	**0.5**	0.3
Clostridiaceae	**0.0**	0.1	**0.0**	0.0	**0.0**	0.0	**0.3**	0.3	*Vagococcus*	**0.4**	0.3	**0.5**	0.4
Conexibacteraceae	**0.0**	0.1	**0.1**	0.1	**0.0**	0.0	**0.3**	0.2	*Epulopiscium*	**0.3**	0.2	**0.5**	0.2
MLE1-12	**0.0**	0.0	**0.0**	0.0	**0.0**	0.0	**0.2**	0.4	*Lactobacillus*	**0.8**	0.6	**0.4**	0.5
Bacillales	**0.1**	0.0	**0.1**	0.2	**0.2**	0.1	**0.2**	0.3	*Ralstonia*	**0.2**	0.2	**0.4**	0.5
Sporosarcina	**0.1**	0.1	**0.1**	0.0	**0.2**	0.1	**0.2**	0.2	*Rikenellaceae*	**1.7**	0.5	**0.4**	0.6
Thermogemmatisporaceae	**0.0**	0.0	**0.0**	0.0	**0.0**	0.0	**0.2**	0.3	*Myroides*	**0.2**	0.2	**0.4**	0.1
Solibacillus	**0.0**	0.0	**0.0**	0.0	**0.0**	0.0	**0.2**	0.4	*Streptococcus*	**0.7**	0.6	**0.4**	0.4

Ruminococcaceae	**0.1**	0.1	**0.1**	0.1	**0.3**	0.0	**0.2**	0.2	*Blautia*	**1.3**	1.7	**0.4**	0.2
Curtobacterium	**0.3**	0.2	**0.3**	0.1	**0.2**	0.1	**0.2**	0.1	*Ruminococcus*	**1.1**	0.5	**0.4**	0.0
Virgibacillus	**0.1**	0.1	**0.1**	0.0	**0.0**	0.0	**0.2**	0.2	*Roseburia*	**0.5**	0.4	**0.4**	0.1
TM7-1	**0.0**	0.0	**0.0**	0.0	**0.0**	0.0	**0.2**	0.3	*Bifidobacterium*	**1.8**	2.8	**0.3**	0.1
Enterococcus	**0.1**	0.1	**0.1**	0.1	**0.2**	0.1	**0.2**	0.2	*Clostridiaceae*	**0.3**	0.1	**0.3**	0.1
Anaerobacillus	**0.0**	0.0	**0.1**	0.1	**0.1**	0.1	**0.2**	0.3	*Eggerthella*	**0.3**	0.2	**0.3**	0.0
Ellin329	**0.0**	0.1	**0.0**	0.0	**0.0**	0.0	**0.2**	0.3	*Collinsella*	**0.5**	0.5	**0.3**	0.2
Sediminibacterium	**0.2**	0.1	**0.1**	0.2	**0.1**	0.1	**0.1**	0.2	*Enhydrobacter*	**0.1**	0.1	**0.3**	0.4
Carnobacterium	**0.1**	0.1	**0.1**	0.1	**0.1**	0.0	**0.1**	0.0	*Prevotella*	**0.2**	0.2	**0.3**	0.4
Methylobacterium	**0.0**	0.0	**0.1**	0.1	**0.0**	0.0	**0.1**	0.2	*Parabacteroides*	**0.7**	0.5	**0.3**	0.5
Ralstonia	**0.1**	0.0	**0.1**	0.1	**0.0**	0.0	**0.1**	0.2	*Bradyrhizobium*	**0.2**	0.2	**0.3**	0.0
WPS-2	**0.0**	0.0	**0.0**	0.0	**0.0**	0.0	**0.1**	0.2	*Corynebacterium*	**0.3**	0.4	**0.3**	0.4
Tissierella_Soehngenia	**0.1**	0.1	**0.0**	0.0	**0.0**	0.0	**0.1**	0.2	*Bacillales*	**0.2**	0.2	**0.3**	0.1
Collinsella	**0.1**	0.1	**0.0**	0.0	**0.2**	0.3	**0.1**	0.1	*Lysinibacillus*	**0.2**	0.2	**0.2**	0.1
Bacteroides	**0.1**	0.1	**0.1**	0.0	**0.1**	0.1	**0.1**	0.1	*Phascolarctobacterium*	**0.4**	0.4	**0.2**	0.2
Planctomyces	**0.0**	0.0	**0.0**	0.0	**0.0**	0.0	**0.1**	0.2	*Paenibacillus*	**0.1**	0.2	**0.2**	0.1
Ochrobactrum	**0.0**	0.0	**0.1**	0.1	**0.1**	0.0	**0.1**	0.1	*Coprococcus*	**0.5**	0.4	**0.2**	0.2
Staphylococcus	**0.2**	0.1	**0.3**	0.2	**0.1**	0.0	**0.1**	0.1	*MLE1-12*	**0.1**	0.1	**0.2**	0.1
Pediococcus	**0.3**	0.1	**0.2**	0.0	**0.0**	0.0	**0.1**	0.1	*Oscillospira*	**0.3**	0.3	**0.2**	0.2
Peptostreptococcus	**0.1**	0.2	**0.0**	0.0	**0.0**	0.0	**0.1**	0.1	*Anaerostipes*	**0.1**	0.2	**0.2**	0.2
Ureibacillus	**0.0**	0.0	**0.0**	0.1	**0.1**	0.1	**0.1**	0.0	*Sphingomonas*	**0.1**	0.1	**0.2**	0.2
Enterobacteriaceae	**0.1**	0.2	**0.1**	0.1	**0.0**	0.0	**0.1**	0.2	*Acinetobacter*	**0.1**	0.1	**0.2**	0.0
JG30-KF-CM45	**0.0**	0.0	**0.0**	0.0	**0.0**	0.0	**0.1**	0.2	*Serratia*	**0.1**	0.1	**0.2**	0.2
Thermicanaceae	**0.0**	0.0	**0.0**	0.0	**0.0**	0.0	**0.1**	0.2	*mitochondria*	**0.1**	0.0	**0.2**	0.1
Rs-045	**0.1**	0.1	**0.1**	0.1	**0.2**	0.1	**0.1**	0.1	*Anaerovorax*	**0.1**	0.1	**0.2**	0.1
Sinobacteraceae	**0.0**	0.0	**0.0**	0.0	**0.1**	0.1	**0.1**	0.1	*Staphylococcus*	**0.1**	0.2	**0.2**	0.2
Brevibacillus	**0.0**	0.0	**0.0**	0.0	**0.0**	0.0	**0.1**	0.1	*Ochrobactrum*	**0.0**	0.0	**0.2**	0.1
Erysipelotrichaceae	**0.0**	0.0	**0.0**	0.0	**0.0**	0.0	**0.1**	0.1	*Parachlamydia*	**0.1**	0.0	**0.1**	0.1
Pseudomonas	**0.0**	0.0	**0.0**	0.1	**0.0**	0.0	**0.1**	0.1	*Thermogemmatisporaceae*	**0.1**	0.2	**0.1**	0.2
Acinetobacter	**0.0**	0.0	**0.1**	0.1	**0.0**	0.0	**0.1**	0.1	*Morganella*	**0.0**	0.0	**0.1**	0.1
Clostridiales	**0.1**	0.0	**0.0**	0.0	**0.2**	0.1	**0.1**	0.1	*Halomonas*	**0.0**	0.1	**0.1**	0.1
Bradyrhizobiaceae	**0.0**	0.0	**0.0**	0.0	**0.0**	0.0	**0.1**	0.1	*Ellin329*	**0.1**	0.1	**0.1**	0.1

Lysinibacillus	**0.0**	0.1	**0.1**	0.1	**0.1**	0.1	**0.1**	0.0
Saccharopolyspora	**0.0**	0.0	**0.1**	0.1	**0.1**	0.0	**0.1**	0.1
Megamonas	**0.0**	0.0	**0.0**	0.0	**0.0**	0.0	**0.1**	0.1
Aneurinibacillus	**0.1**	0.1	**0.0**	0.0	**0.0**	0.0	**0.1**	0.0
Oxalobacteraceae	**0.0**	0.0	**0.0**	0.0	**0.0**	0.0	**0.1**	0.1
Akkermansia	**0.0**	0.1	**0.0**	0.0	**0.0**	0.0	**0.1**	0.1
Agrobacterium	**0.0**	0.0	**0.0**	0.0	**0.0**	0.0	**0.1**	0.1
Kurthia	**0.3**	0.1	**0.1**	0.1	**0.2**	0.1	**0.1**	0.1
Myroides	**0.0**	0.0	**0.1**	0.0	**0.1**	0.1	**0.1**	0.0
Microbacterium	**0.1**	0.0	**0.0**	0.0	**0.1**	0.0	**0.1**	0.1
Micrococcaceae	**0.0**	0.0	**0.0**	0.0	**0.0**	0.0	**0.1**	0.1
Micrococcus	**0.0**	0.0	**0.0**	0.0	**0.0**	0.0	**0.1**	0.1
Mycetocola	**0.1**	0.1	**0.1**	0.1	**0.1**	0.0	**0.1**	0.0
Halomonas	**0.0**	0.0	**0.0**	0.0	**0.0**	0.0	**0.1**	0.1
Coriobacteriaceae	**0.1**	0.1	**0.1**	0.2	**0.1**	0.0	**0.1**	0.1
Ruminococcus	**0.0**	0.0	**0.1**	0.1	**0.0**	0.0	**0.1**	0.0
Frigoribacterium	**0.1**	0.0	**0.0**	0.0	**0.0**	0.0	**0.0**	0.1
Streptomyces	**0.1**	0.1	**0.0**	0.1	**0.0**	0.0	**0.0**	0.1
Tepidimicrobium	**0.0**	0.0	**0.1**	0.0	**0.0**	0.0	**0.0**	0.1
Peptoniphilus	**0.1**	0.2	**0.0**	0.1	**0.0**	0.0	**0.0**	0.0
Vagococcus	**0.1**	0.0	**0.1**	0.1	**0.0**	0.0	**0.0**	0.0
Brevibacterium	**0.0**	0.0	**0.0**	0.1	**0.1**	0.1	**0.0**	0.0
Tissierellaceae	**0.1**	0.1	**0.0**	0.0	**0.0**	0.0	**0.0**	0.0
Paracoccus	**0.3**	0.4	**0.1**	0.1	**0.0**	0.1	**0.0**	0.0
Enhydrobacter	**0.3**	0.5	**0.0**	0.0	**0.0**	0.0	**0.0**	0.0
Planococcaceae	**0.0**	0.0	**0.1**	0.0	**0.0**	0.0	**0.0**	0.0
Paraprevotella	**0.0**	0.0	**0.1**	0.2	**0.0**	0.0	**0.0**	0.0
Roseomonas	**0.2**	0.3	**0.0**	0.0	**0.0**	0.0	**0.0**	0.0
Unassigned	**0.1**	0.1	**0.1**	0.0	**0.1**	0.0	**0.2**	0.2

Stenotrophomonas	**0.1**	0.1	**0.1**	0.1
Jeotgalicoccus	**0.1**	0.1	**0.1**	0.2
Allobaculum	**0.1**	0.1	**0.1**	0.1
Ureibacillus	**0.1**	0.1	**0.1**	0.0
Pseudomonas	**0.1**	0.2	**0.1**	0.1
Leuconostocaceae	**0.1**	0.0	**0.1**	0.1
Fusobacterium	**0.1**	0.1	**0.1**	0.0
Parachlamydiaceae	**0.0**	0.0	**0.1**	0.1
Christensenellaceae	**0.3**	0.0	**0.1**	0.1
Solibacterales	**0.1**	0.1	**0.1**	0.1
Sutterella	**0.4**	0.4	**0.1**	0.1
Natronobacillus	**0.0**	0.0	**0.1**	0.1
Sinobacteraceae	**0.0**	0.0	**0.1**	0.1
Dietzia	**0.1**	0.1	**0.1**	0.1
Paracoccus	**0.0**	0.1	**0.1**	0.1
Rhodococcus	**0.1**	0.1	**0.1**	0.0
Lachnospiraceae;Other	**0.0**	0.1	**0.1**	0.0
Dechloromonas	**0.0**	0.0	**0.1**	0.1
Aneurinibacillus	**0.1**	0.1	**0.1**	0.1
RF32	**0.1**	0.1	**0.1**	0.1
Enterococcus	**0.0**	0.0	**0.1**	0.0
Prevotella	**0.0**	0.2	**0.1**	0.4
MND1	**0.0**	0.0	**0.1**	0.1
Barnesiellaceae	**0.2**	0.2	**0.1**	0.1
Sporanaerobacter	**0.1**	0.2	**0.1**	0.1
Actinomyces	**0.1**	0.1	**0.1**	0.1
Ellin6513	**0.0**	0.0	**0.1**	0.0
AF12	**0.0**	0.0	**0.1**	0.1
Rubellimicrobium	**0.0**	0.0	**0.1**	0.1
Chryseobacterium	**0.0**	0.1	**0.1**	0.1
Rhodocyclaceae	**0.0**	0.0	**0.1**	0.1
Tepidimicrobium	**0.1**	0.1	**0.1**	0.1

Pseudoramibacter_Eubacterium	**0.1**	0.0	**0.1**	0.0
Pseudomonadaceae	**0.0**	0.0	**0.1**	0.0
Conexibacteraceae	**0.0**	0.0	**0.1**	0.1
Pirellulaceae	**0.0**	0.0	**0.1**	0.1
Butyricimonas	**0.0**	0.0	**0.1**	0.0
Mycobacterium	**0.0**	0.0	**0.1**	0.1
Paludibacter	**0.1**	0.0	**0.0**	0.0
Thermoactinomyces	**0.1**	0.1	**0.0**	0.0
Ruminococcus	**0.3**	0.5	**0.0**	0.0
Anaerotruncus	**0.1**	0.1	**0.0**	0.1
Luteimonas	**0.1**	0.1	**0.0**	0.1
Dialister	**0.5**	0.8	**0.0**	0.0
Acidaminococcus	**0.1**	0.1	**0.0**	0.0
Tissierellaceae	**0.1**	0.0	**0.0**	0.0
Turicibacter	**0.1**	0.1	**0.0**	0.0
Odoribacter	**0.1**	0.1	**0.0**	0.0
Bilophila	**0.3**	0.5	**0.0**	0.0
Catenibacterium	**0.1**	0.2	**0.0**	0.0
Anaerobiospirillum	**0.2**	0.3	**0.0**	0.0
Slackia	**0.1**	0.2	**0.0**	0.0
Megamonas	**0.1**	0.1	**0.0**	0.0
Other	**1.3**	1.2	**1.2**	1.3

Supplementary Table S2. Statistical analysis on gut microbiota detected in the hindgut content of European sea bass fed different diets at 71 days (pre-suboptimal rearing condition), and at 81 days (post-suboptimal rearing condition). All the *P* values were appropriately corrected for multiple comparisons using the Benjamini-Hochberg method. False discovery rate (FDR) ≤ 0.05 was considered as statistically significant. D0= 0 mg Kg $^{-1}$ organic acids and and nature-identical compounds (OA, NIC) ; D250 = 250 mg Kg $^{-1}$OA, NIC; D500 = 500 mg Kg $^{-1}$ OA, NIC; D1000 = 1000mg Kg $^{-1}$ OA, NIC. D0vsD250=comparisons between D0 and D250 genera under pre-suboptimal rearing condition; D0vsD500=comparisons between D0 and D500 genera under pre-suboptimal rearing condition; D0vsD1000=comparisons between D0 and D1000 genera under pre-suboptimal rearing condition and post-suboptimal rearing condition; D0vsD0=comparisons between pre-suboptimal rearing condition and post- suboptimal rearing condition D0 genera; D1000vsD1000=comparisons between pre- suboptimal rearing condition and post- suboptimal rearing condition D1000 genera.

	**Pre-suboptimal rearing condition**			**Post-suboptimal rearing condition**		**Pre-Post suboptimal rearing condition**		
**Diet**	**D0 vs D250**	**D0 vs D500**	**D0 vs D1000**		**D0 vs D1000**		**D0 vs D0**	**D1000 vs D1000**
**Genus**	P- value				P-value		P-value	
Lactobacillus	0.66	0.08	0.66	*Enterobacteriaceae*	1.0	*Rikenellaceae*	0.06	0.08
Leuconostocaceae	0.08	0.08	0.66	*Eubacterium*	0.7	*Myroides*	0.18	0.1
Streptococcus	1.0	0.66	1.0	*Bacillus*	0.7	*Acinetobacter*	0.06	0.7
Bacillus	1.0	1.0	0.38	*Clostridium*	0.7	*Enterococcus*	0.08	0.7
Corynebacterium	0.08	0.19	0.08	*Erysipelotrichaceae*	0.4	*Planctomyces*	0.2	0.5
Leuconostoc	0.12	0.66	1.0	*Bacteroides*	0.2	*Paraprevotella*	0.64	NA
Bacillaceae	0.66	0.38	0.38	*Coriobacteriaceae*	1.0	*Christensenellaceae*	0.08	0.06
Streptophyta	0.66	0.19	0.08	*Lachnospiraceae*	1.0	*Morganella*	0.18	0.06
Geobacillus	0.38	0.38	0.38	*Mogibacteriaceae*	0.4	*Akkermansia*	0.08	0.08
mitochondria	0.66	0.19	0.38	*Bacillaceae*	0.4	*Ruminococcaceae*	0.08	0.1
Lactococcus	0.19	0.38	0.66	*Peptostreptococcaceae*	0.4	*Bacteroides*	0.1	0.1
Bifidobacteriaceae	0.82	0.66	1.0	*Ruminococcaceae*	0.2	*Streptophyta*	0.1	0.2
Clostridium	1.0	0.38	0.27	*Geobacillus*	1.0	*Sinobacteraceae*	0.38	1.0
Bifidobacterium	1.0	1.0	0.66	*Dorea*	1.0	*Propionibacteriaceae*	0.08	0.38
Bradyrhizobium	1.0	0.82	0.38	*Sediminibacterium*	1.0	*Lactobacillales*	0.08	0.08
Delftia	0.66	1.0	0.38	*Caloramator*	1.0	*Leuconostoc*	0.08	0.08
Peptostreptococcaceae	1.0	0.26	0.66	*Carnobacterium*	0.7	*Legionellaceae*	1.0	0.5
Caulobacteraceae	0.66	0.82	0.66	*Clostridiales*	0.7	*Natronobacillus*	1.0	0.51

Faecalibacterium	0.37	0.64	0.66
Sphingobium	0.64	0.2	0.06
Rhodococcus	1.0	0.66	0.19
Lactobacillales	0.08	0.08	0.82
Sporanaerobacter	0.66	1.0	0.38
Blautia	1.0	0.38	0.66
Solibacterales	0.38	1.0	1.0
Sphingomonas	1.0	0.82	1.0
Ellin6513	1.0	0.38	0.66
Ruminococcus	0.38	0.66	0.08
Lachnospiraceae	1.0	1.0	0.66
Lactobacillaceae	0.38	1.0	1.0
Clostridiaceae	1.0	0.66	0.18
Conexibacteraceae	0.64	0.64	0.08
Bacillales	1.0	0.27	0.38
Sporosarcina	0.66	0.65	0.38
Ruminococcaceae	1.0	0.08	1.0
Curtobacterium	1.0	0.38	0.66
Enterococcus	1.0	0.19	0.82
Anaerobacillus	0.5	0.08	1.0
Sediminibacterium	1.0	0.51	1.0
Carnobacterium	0.51	0.82	1.0
Ralstonia	0.82	0.51	1.0
Collinsella	0.5	1.0	0.38
Bacteroides	0.38	0.27	1.0
Ochrobactrum	0.66	0.27	0.66
Staphylococcus	0.38	0.51	0.66
Pediococcus	0.35	0.08	0.08
Ureibacillus	0.66	0.66	0.12
Enterobacteriaceae	1.0	1.0	1.0
Rs-045	0.65	0.19	0.65
Sinobacteraceae	0.81	0.66	0.35

Bacteroides	0.2
Delftia	1.0
Sphingobium	0.7
Anaerobacillus	0.4
Lachnospira	0.4
Streptophyta	0.2
Faecalibacterium	1.0
Caulobacteraceae	0.5
Akkermansia	0.2
Methylobacterium	0.7
Vagococcus	1.0
Lactobacillus	0.4
Ralstonia	1.0
Rikenellaceae	0.1
Myroides	0.1
Streptococcus	0.5
Blautia	0.7
Ruminococcus	0.4
Roseburia	0.7
Bifidobacterium	1.0
Clostridiaceae	0.7
Eggerthella	0.7
Collinsella	1.0
Enhydrobacter	1.0
Prevotella	0.2
Parabacteroides	0.4
Bradyrhizobium	0.7
Corynebacterium	1.0
Bacillales	0.16
Lysinibacillus	1.0
Phascolarctobacterium	0.0
Paenibacillus	0.66

mitochondria	0.08	0.1
Ellin6513	0.07	1.0
Coprococcus	0.1	0.06
Mogibacteriaceae	0.08	0.06
Parachlamydia	0.06	0.06
Lachnospira	0.06	0.06
Anaerobacillus	0.08	0.08
Erysipelotrichaceae	0.1	0.1
Lactobacillus	0.1	0.1
Peptostreptococcaceae	0.1	0.1
Parabacteroides	0.08	0.2
Ochrobactrum	1.0	0.7
Ruminococcus	0.1	1.0
Ruminococcus	0.08	1.0
Virgibacillus	0.07	0.08
Curtobacterium	0.1	0.08
Comamonadaceae	1.0	0.82
Adlercreutzia	0.2	1.0
Solibacillus	0.2	1.0
Janthinobacterium	0.64	1.0
Rubellimicrobium	0.19	0.35
Dechloromonas	0.65	0.38
Megamonas	0.5	0.2
Brevibacillus	0.65	0.2
Peptostreptococcus	0.35	0.2
Pediococcus	0.06	0.35
Tissierella_Soehngenia	0.06	0.35
Lactobacillaceae	0.06	0.35
Myxococcales	0.2	0.35
Jonesiaceae	0.5	0.5
Slackia	0.5	0.5
Trichococcus	0.5	0.5

Clostridiales	1.0	0.19	0.66
Bradyrhizobiaceae	1.0	0.82	0.38
Lysinibacillus	0.35	0.48	0.5
Saccharopolyspora	0.82	0.81	0.66
Oxalobacteraceae	0.82	0.51	1.0
Kurthia	0.08	0.38	0.08
Coriobacteriaceae	0.66	0.65	0.38
Streptomyces	0.27	0.27	0.27
Vagococcus	0.82	0.18	0.12
Tissierellaceae	0.66	1.0	0.38
Unassigned	0.18	0.26	1.0

Coprococcus	0.4
MLE1-12	0.7
Oscillospira	1.0
Anaerostipes	0.82
Sphingomonas	0.82
Acinetobacter	0.1
mitochondria	0.38
Staphylococcus	0.66
Ochrobactrum	0.4
Parachlamydia	0.4
Thermogemmatisporaceae	1.0
Morganella	0.2
Halomonas	0.51
Ellin329	1.0
Stenotrophomonas	0.51
Jeotgalicoccus	1.0
Ureibacillus	0.51
Pseudomonas	1.0
Leuconostocaceae	1.0
Parachlamydiaceae	0.4
Christensenellaceae	0.2
Solibacterales	1.0
Natronobacillus	0.35
Sinobacteraceae	0.2
Dietzia	0.82
Paracoccus	0.7
Rhodococcus	0.66
Lachnospiraceae;Other	0.65
Dechloromonas	0.5
Aneurinibacillus	1.0
Enterococcus	0.16
Prevotella	0.2

Roseomonas	0.5	0.5
Shewanella	0.5	0.5
TM7-1	0.5	0.5
Arthrobacter	0.64	0.5
Planococcaceae	NA	0.5
ZB2	NA	0.5
Brevundimonas	NA	0.5
Rhodoplanes	NA	0.5
Phyllobacterium	NA	0.5
Edaphobacter	NA	1.0
Devosia	NA	1.0
Acetobacteraceae	NA	1.0
Nocardioidaceae	0.5	1.0
Anaerococcus	0.5	1.0
Agrobacterium	0.5	1.0
0319-6G20	0.5	1.0
Rummeliibacillus	0.08	NA
Solirubrobacterales	0.5	NA
Gemmata	0.5	NA
Gluconacetobacter	0.5	NA
Stenotrophomonas	0.35	0.08
Halomonas	0.35	0.38
Tissierellaceae	1.0	0.64
Ureibacillus	0.7	1.0
Caldicoprobacter	0.35	0.19
Conexibacteraceae	0.37	0.1
Lactococcus	0.08	0.1
SJA-4	0.2	0.5
Barnesiellaceae	0.2	1.0
Turicibacter	1.0	1.0
Lachnospiraceae;Other	0.5	0.07
Sporosarcina	0.38	0.1

Barnesiellaceae	0.64
Sporanaerobacter	1.0
Actinomyces	1.0
Ellin6513	0.38
Rubellimicrobium	0.5
Chryseobacterium	1.0
Rhodocyclaceae	0.5
Tepidimicrobium	1.0
Pseudoramibacter_Eubacterium	1.0
Pseudomonadaceae	1.0
Conexibacteraceae	0.64
Pirellulaceae	1.0
Ruminococcus	0.4
Acidaminococcus	1.0
Tissierellaceae	0.51
Turicibacter	0.64
Bilophila	1.0
Slackia	0.5
Megamonas	0.5

Bacillaceae;Other	0.64	0.82
Anoxybacillus	1.0	0.06
Rhodococcus	0.1	0.08
Paenibacillus	1.0	0.08
Bradyrhizobium	1.0	0.66
Staphylococcus	0.66	1.0
Paracoccus	0.82	0.06
Eggerthella	0.2	0.06
Roseburia	0.06	0.08
Eubacterium	0.08	0.08
Clostridium	0.1	0.1
Clostridiales	0.1	0.1
Carnobacterium	0.1	0.1
Methylobacterium	0.2	0.2
Sphingobium	0.06	0.4
Bacillus	1.0	0.7
MLE1-12	0.18	0.7
Blautia	0.4	0.7
Novosphingobium	0.35	0.19
Erwinia	0.5	0.2
Peptoniphilus	0.5	1.0
Phascolarctobacterium	0.35	0.06
Anaerostipes	0.35	0.2
Dietzia	0.18	0.64
Sphingomonas	0.7	1.0
Enhydrobacter	1.0	0.06
Pseudoramibacter_Eubacterium	0.06	0.06
Oscillospira	0.06	0.06
Vagococcus	0.1	0.08
Lysinibacillus	0.18	0.08
Caloramator	0.06	0.08
Dorea	0.1	0.08

Bifidobacteriaceae	0.08	0.08
Corynebacterium	0.1	0.1
Sporanaerobacter	0.37	0.1
Coriobacteriaceae	0.1	0.1
Lachnospiraceae	0.08	0.1
Leuconostocaceae	0.1	0.1
Bifidobacterium	1.0	0.1
Enterobacteriaceae	0.08	0.1
Streptococcus	0.1	0.1
Sediminibacterium	0.4	0.1
Alkaliphilus	0.64	0.2
Other	0.08	0.2
WPS-2	1.0	0.35
Prevotella	0.06	0.35
Micrococcaceae	0.64	0.35
Leucobacter	0.35	0.35
Microbacterium	0.07	0.35
Microbacteriaceae	0.18	0.35
Collinsella	0.4	0.4
Delftia	0.7	0.4
Solibacterales	0.4	0.4
Ralstonia	0.7	0.4
Veillonella	0.64	0.5
Rothia	1.0	0.5
Propionibacterium	1.0	0.5
Actinomycetales	0.5	0.5
Chryseobacterium	0.5	0.5
Pirellulaceae	0.5	0.5
Kurthia	0.1	0.51
Rhizobiales	1.0	0.51
Tepidimicrobium	0.7	0.64
Ellin329	0.35	0.66

Clostridiaceae	0.08	0.7
Bacillales	0.7	0.7
Geobacillus	0.7	0.7
Faecalibacterium	0.18	0.7
Caulobacteraceae	0.7	0.7
Pseudomonas	1.0	1.0
Bacillaceae	0.7	1.0
Kocuria	0.35	1.0
Oxalobacteraceae	0.49	1.0
Acidaminococcus	0.5	1.0
Bradyrhizobiaceae	0.5	1.0
Phyllobacteriaceae	0.5	1.0
Bilophila	0.5	1.0
Thermogemmatisporaceae	0.82	1.0
Aneurinibacillus	0.7	1.0
Actinomyces	1.0	1.0
Brevibacterium	1.0	1.0
Cloacibacterium	1.0	1.0
Jeotgalicoccus	1.0	1.0
Gemmataceae	1.0	1.0
Mycetocola	0.06	0.06
Rs-045	0.06	0.06
Streptomyces	0.06	0.2
Facklamia	0.5	0.2
Rathayibacter	0.06	0.2
Saccharopolyspora	0.06	0.2
Frigoribacterium	0.06	0.2
Sanguibacter	0.2	0.5
Agrococcus	NA	0.5
JG30-KF-CM45	NA	0.5
Paenibacillaceae	NA	0.5
Erysipelothrix	NA	0.5

		Isosphaeraceae	NA	0.5
		Macrococcus	0.2	0.5
		Williamsia	0.5	0.5
		Photobacterium	0.5	0.5
		Weissella	0.19	NA
		Cellulomonas	0.5	NA
		Proteiniclasticum	0.5	NA
		Peptococcus	0.5	NA
